# Identification of Target Genes Using Innovative Screening Systems

**DOI:** 10.1111/pin.70019

**Published:** 2025-05-05

**Authors:** Keisuke Sugita, Morito Kurata

**Affiliations:** ^1^ Department of Comprehensive Pathology, Graduate School of Medical and Dental Sciences Institute of Science Tokyo Tokyo Japan; ^2^ Department of Pathology, The Cancer Institute Hospital Japanese Foundation for Cancer Research Tokyo Japan; ^3^ Division of Pathology, The Cancer Institute Japanese Foundation for Cancer Research Tokyo Japan; ^4^ Pathology, Division of Integrated Facilities, Hospital Institute of Science Tokyo Tokyo Japan

**Keywords:** CRISPR screening, drug resistance, oncogene, random mutagenesis, reverse genetic screening

## Abstract

Advances in cancer biology have been achieved by the identification of oncogenes and tumor suppressor genes through the remarkable progression of next‐generation sequencing. New techniques, such as single‐cell analysis, help uncover cancer progression and heterogeneity. Reverse genetic screenings, including methods like random mutagenesis via retroviruses, transposons, RNA interference, and CRISPR, are useful for exploring gene functions and their roles in cancer. Especially in random mutagenesis, CRISPR screening and its modifications have recently emerged as powerful tools due to their comprehensiveness and simplicity in inducing genetic mutations. Initially, CRISPR screening focused on analyzing biological phenotypes in a cell population. It has since evolved to incorporate advanced techniques, such as combining single‐cell and spatial analyses. These developments enable the investigation of cell–cell and spatial interactions, which more closely mimic In Vivo microenvironments, offering deeper insights into complex biological processes. These approaches allow for the identification of essential genes involved in cancer survival, drug resistance, and tumorigenesis. Together, these technologies are advancing cancer research and therapeutic development.

**Abbreviations**
3D: three dimensions
ALL: acute lymphoblastic leukemia
BCR: B‐cell receptor
BLNK: B‐cell linker
BMS: Bristol Myers Squibb
c/ebpb: CCAAT/enhancer‐binding protein beta
CaRPool: Cas13 RNA perturb
Cas: CRISPR‐associated protein
CIS: common integration sites
CITE: cellular indexing of transcriptomes and epitopes
CRISPR: clustered regularly interspaced short palindromic repeats
CRISPRa: CRISPR activation
CRISPRi: CRISPR interference
CROP: CRISPR droplet
Dck: Deoxycytidine Kinase
Depmap: dependency map
EGFR: epidermal growth factor receptor
FACS: fluorescence‐activated cell sorter
gRNA: guide RNA
HEK: human embryonic kidney cell
KRAS: Kirsten ras proto‐oncogene, GTPase
LTR: long terminal repeat
M1AP: meiosis 1 associated protein
MIMOSCA: multiple input multiple output single‐cell analysis
MOI: multiplicity of infection
mRNA: messenger RNA
MYC: myelocytomatosis oncogene
NF‐κB: nuclear factor‐kappa B
NGS: next‐generation sequencing
PICKLES: pooled in vitro CRISPR knockout library essentiality screens
Pro‐Code: protein barcoding
Rhbdd2: rhomboid domain containing 2
RIM: retroviral insertional mutagenesis
RNAi: RNA interference
SA: splice acceptor
Scifi: single‐cell combinatorial fluidic indexing
scRNA: single‐cell RNA
Seq: sequencing
shRNA: short hairpin RNA
SPEAC: systematic perturbation of encapsulated associated cells
TCGA: The Cancer Genome Atlas
TCR: T‐cell receptor
Tgfbr2: transforming growth factor, beta receptor 2
TGFβ: transforming growth factor beta
TILs: tumor‐infiltrating lymphocytes
TME: tumor microenvironment
TSS: transcription start site
UV: ultraviolet

## 1 Introduction

Clarifying biological processes is crucial for advancing life sciences. In particular, in cancer biology, identifying oncogenes and tumor suppressor genes as therapeutic targets is driving medical progress. In recent decades, extensive sequencing of cancer genomics, such as The Cancer Genome Atlas (TCGA) [1] (http://cancergenome.nih.gov/abouttcga), has rapidly developed alongside next‐generation sequencing (NGS), enabling deep analysis of cancer genomics. Furthermore, single‐cell analysis has become widely used in recent studies, revealing the progression and heterogeneity of cancer tissues. The process of reading the genome sequences of cancer to identify genes responsible for carcinogenesis is known as “forward genetic screening,” which has contributed significantly to recent scientific advancements. Forward genetic screenings can reveal a large number of mutated genes; however, since they are observational analyses, not all detected mutated genes are oncogenes or tumor suppressor genes. Both driver genes and passenger genes are identified simultaneously, and further functional validation is required to confirm their roles.

On the other hand, reverse genetic screening is widely used to explore biological mechanisms through functional screening. In this method, random mutagenesis is introduced into cells, which then undergo changes in cellular phenotypes, such as altered cell proliferation or drug resistance. Various agents for inducing random mutagenesis have been reported. The present review article will examine the utility of these random mutagenesis systems as genetic perturbations in functional screening.

### 1.1 The Evolution of Random Mutation as a Genetic Perturbation

Well‐established screening methods of random mutagenesis include chemical carcinogenesis, retroviruses, and transposons. Additionally, RNA interference libraries have greatly improved specificity and simplicity compared to conventional screening methods by employing gene barcodes. Over the past decade, CRISPR technology has also advanced significantly. Each of these techniques has its own advantages and disadvantages, as shown in Table 1.

**Table 1:** Advantages and disadvantages of mutagenesis.

Types	Basic principle	Advantages	Disadvantages
Retrovirus	Gene activation and knockout	Strong gene activation by long terminal repeats (LTR)	Low efficiency for tumor suppressor genes. Identification methods are complicated.
Transposon	Gene activation and knockout	Strong gene activation by strong promoter elements In Vivo screening with tissue‐specific mutagenesis	Identification methods are complicated.
shRNA library	Knockdown	Convenient methods using barcode sequence	Comparable with CRISPR screening.
CRISPR library	Knockout Activation Knockdown	Convenient methods Only for knockout Only for gene activation Using CRISPR interference (CRISPRi)	The In Vivo model has limitations. Random mutations inducted cells can be transplanted In Vivo. However, tissue‐specific random mutagenesis is difficult.

The identification of carcinogenic genes using chemical carcinogenesis was previously considered a labor‐intensive process. However, with recent advancements in NGS, tumor samples induced by chemical agents can now be easily analyzed using this method to identify carcinogen‐responsible genes. This enables more efficient combination studies. Below are some common examples of mutagenic (or mutagen‐like) conditions for further review.

#### 1.1.1 Retrovirus

**Figure 1:**
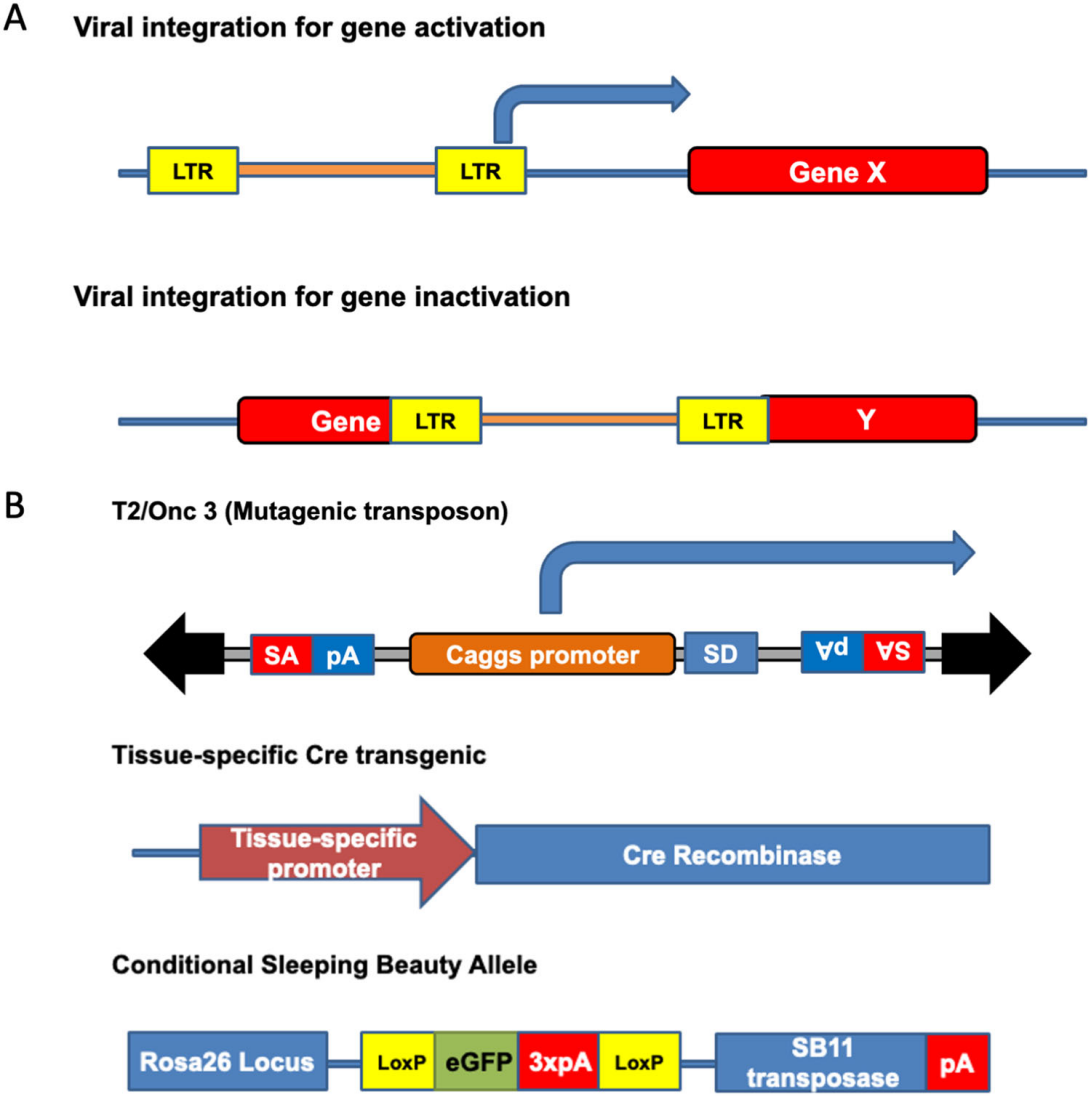
Gene activation and interruption by the retrovirus and transposon. (A) Long terminal repeats (LTR) of retrovirus activates nearby gene expression. Gene inactivation can be induced by the retrovirus integration in the coding gene. (B) Integrated transposon can gene activation by the strong promoter such as CMV‐enhancer–chicken beta‐actin (CAGGS) promoter. Transposon can be jumping and moving randomly in the genomic DNA by the transposase. In the mouse model, tissue‐specific promoters activate Cre recombinase and delete the LoxP sandwiched sequence then active transposase. Activated transposase, such as Sleeping Beauty, can let transposons jump and move by cutting and pasting.

Infected retroviruses can integrate into genomic DNA, and their long terminal repeat (LTR) sequences can activate nearby gene expression at the integration sites, acting as a promoter (Figure 1A). Additionally, when a retrovirus integrates with the coding region of a target gene, it can cause disruption, resulting in loss of function. However, the frequency of such disruptions is low, as the likelihood of retroviral integration occurring in both alleles of the same gene is rare. Retroviral insertional mutagenesis (RIM) is a well‐established method for identifying genes responsible for specific functions.

RIM can be applied to both in vitro and In Vivo models, particularly in mouse models. For example, random mutations are introduced into hematopoietic cells and transplanted to the recipient mice to assess whether these mice develop leukemia. Once leukemia develops, tumor cells are harvested, and viral integration sites can be analyzed to identify the affected genes of leukemogenesis. By analyzing multiple tumors, common integration sites (CIS) are often associated with genes that play key roles in cancer development.

In the process of leukemogenesis, a single gene mutation may increase the risk of tumor development. In fact, in clinical cases, additional environmental factors or genetic mutations contribute to leukemia progression. To explore these cooperative genetic interactions with a single mutation, RIM is often used. For instance, in our previous study of B‐cell acute lymphoblastic leukemia (B‐ALL), B‐cell linker (BLNK) molecule (also known as SLP‐65 or BASH), a cytoplasmic adaptor protein involved in B‐cell receptor signaling, was investigated. While *blnk* deficiency alone takes a long period of time to induce leukemia in mice, additional genetic mutations are required for the development of B‐ALL. By introducing RIM, cooperative effects with CCAAT/enhancer‐binding protein beta (*Cebpb*) were identified, highlighting the complex interactions involved in leukemia development [2].

#### 1.1.2 Transposon

Random mutation analysis using transposons is similar to gene activation with retroviruses, but with the added advantage of stronger, modified promoters, such as the CMV‐enhancer–chicken beta‐actin (CAGGS) promoter used in T2/Onc3 transposon (Figure 1B). This method can also be used for the comprehensive screening of tumor suppressor genes, as the splice acceptor in the transposon reduces gene expression when integrated into target genes, allowing for a larger number of integrations compared to retroviruses.

To activate transposons, transposases such as “Sleeping Beauty” are required to enable the transposons to “jump” randomly to different loci in the genome. Additionally, the transposon system allows for organ‐specific random mutagenesis. Organ‐specific promoters can be used to induce Cre‐recombinase, which, when activated, triggers the transposase at the Rosa locus, enabling the transposon to move randomly and induce mutagenesis in a specific organ. CIS are crucial in transposon screening.

One of the key advantages of the Sleeping Beauty system is its ability to perform In Vivo screening, making it particularly useful for studying genetic mutations in living organisms [3].

#### 1.1.3 RNA Interference (RNAi) Library

**Figure 2:**
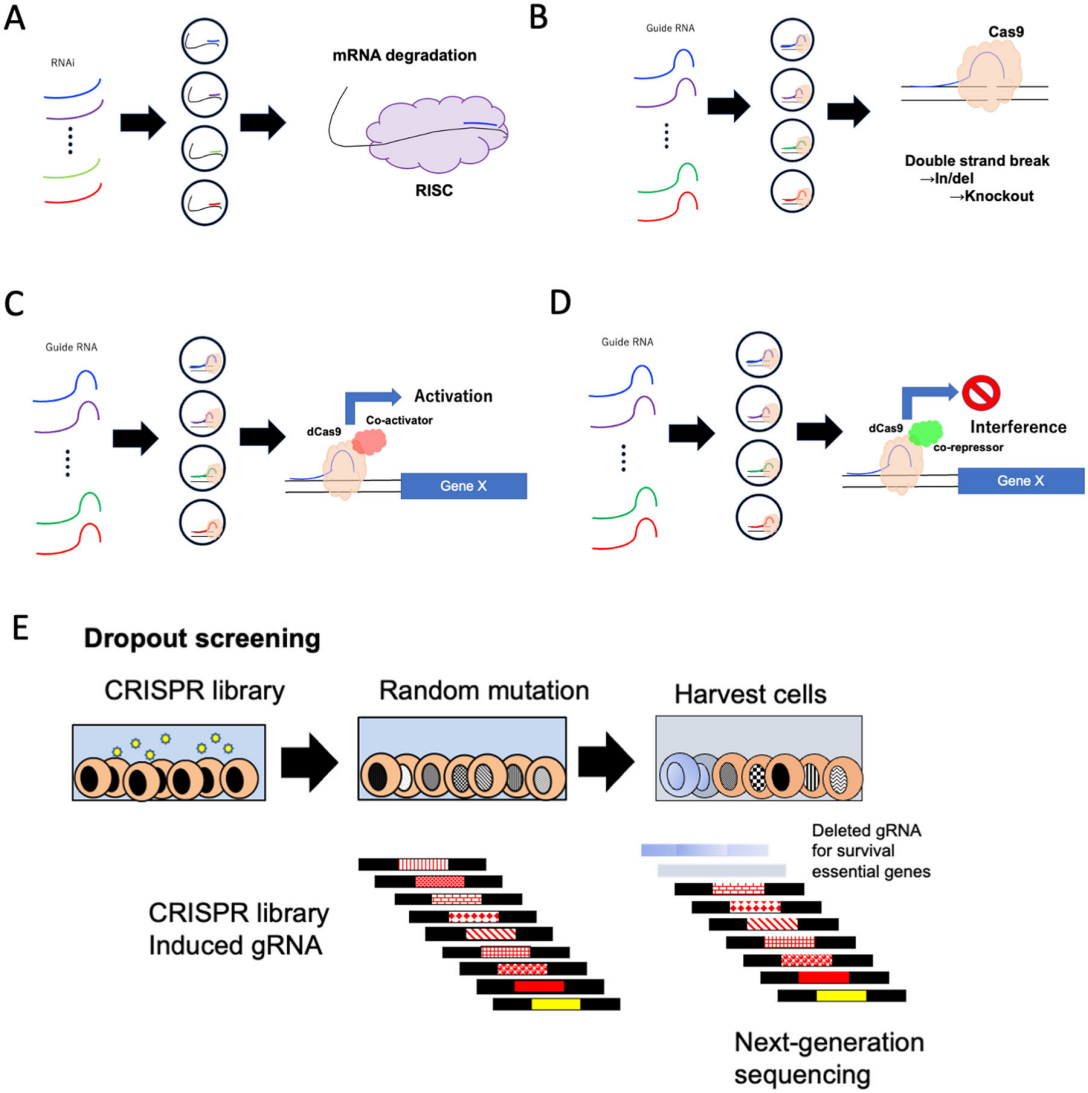
Schemes of clustered regularly interspaced short palindromic repeat (CRISPR) libraries. (A) RNAi library. (B) CRISPR library for knockout. (C) CRISPR activation library. (D) CRISPR interference library. (E) Principle of dropout screening. Depleted guide RNA can be identified by the subtraction of the data sets of next‐generation sequencing (NGS).

RNAi is a widely used and well‐established method used to downregulate specific target genes (Figure 2A), and the short hairpin RNA (shRNA) library is a more sophisticated method of identifying candidate genes for loss‐of‐function screenings, although it cannot activate any gene expression directly. Targeted shRNA can be easily identified, especially when a barcode is encoded. In addition, shRNA libraries have a powerful screening method for identifying candidates in various fields [4, 5].

#### 1.1.4 CRISPR Library

The CRISPR‐associated nuclease Cas9 can introduce loss‐of‐function mutations at specific sites within the genome [6, 7]. Cas9 induces DNA double‐strand breaks at specific genomic loci using synthetic single‐guide RNAs (gRNAs), enabling the generation of frameshift insertion/deletion (in/del) mutations that result in loss‐of‐function alleles (Figure 2B). Specific gRNA sequences can be synthesized at scale through array‐based oligonucleotide library synthesis. This enables pooled genome‐scale functional screening using Cas9. Additionally, CRISPR activation (CRISPRa) and CRISPR interference (CRISPRi) have been developed. These methods use short gRNAs designed upstream of a transcription start site in combination with modified Cas9 proteins fused to a coactivator (for CRISPRa) or a suppressor (for CRISPRi) (Figure 2C,D).

CRISPR libraries are invaluable tools for random mutation screening. Knockout libraries, in particular, are commonly used to identify genes responsible for drug resistance in proliferating cell populations under drug exposure or to discover oncogenes essential for survival in diminishing cell populations. This approach, known as dropout (negative) screening, identifies genes critical for survival under specific conditions. For example, if a cell carries a gRNA targeting a survival‐essential gene, the knockout cell will not proliferate. Cells that survive are collected, and their gRNA pools are sequenced using NGS to compare the initial gRNA pool with the surviving pool. This process identifies survival‐essential candidate genes (Figure 2E). The use of lentiviral delivery facilitates this process, as it allows for controlled multiplicity of infection (MOI) and ensures stable genomic integration during cell replication.

Furthermore, databases such as PICKLES, pooled in vitro CRISPR knockout library essentiality screens [8], (https://pickles.hart-lab.org/) and Depmap [9] (https://depmap.org/portal/) are useful for assessing gene essentiality in cell proliferation. These resources contain data from various cell lines, enabling researchers to design virtual experiments to identify essential genes. In conventional CRISPR screening, candidate genes for drug resistance are identified by tracking increases or decreases in tumor cell populations containing gRNAs that induce random mutations under specific screening conditions, such as drug exposure.

### 1.2 Innovative CRISPR Screening System

#### 1.2.1 Avoiding Picking Up Well‐Known Target Genes in Genome‐Wide Screening

Genome‐wide screening is one of the advantages of CRISPR. However, to find other new mechanisms, that is, avoiding picking up well‐known target genes, modulation of the screening system is needed. For example, *Deoxycytidine kinase* (*Dck*) is well‐known as a drug‐resistant gene for cytarabine. Without library modulation, CRISPR screening reveals that randomly mutated cells can develop resistance to cytarabine, identifying *Dck* as a gene associated with drug resistance. Therefore, we made synonymous‐mutant *Dck* that cannot be recognized by the gRNAs in the CRISPR library. Eventually, a second mechanism for cytarabine resistance is addressed [10].

#### 1.2.2 Finding the Elements in Promoter Complex

In our previous study, we used CRISPR screenings to explore molecules affecting the promoter region of a specific gene and control transcriptional activity. That study aimed to identify new myelocytomatosis oncogene (MYC) transcriptional activators using a CRISPRa library and novel promoter‐reporter systems. We developed a system to evaluate MYC activity and address autofluorescence issues in screening by employing the photoconversion protein Dendra2, which can convert its fluorescence from green to red using UV light (405 nm). The MYC promoter‐reporter system, named pMYC‐promoter‐Dendra2, includes a proximal MYC promoter (3.1 kb). HEK293T cells were transfected with both the CRISPR library and pMYC‐promoter‐Dendra2, and Dendra2‐positive cells, indicating MYC upregulation, were sorted. Out of 169 collected cells, 12 clones were established, with two showing Dendra2 positivity upon re‐transfection. Eventually, only one gene, *M1AP* was confirmed to induce high MYC expression and a significant increase of promoter activity of MYC [11, 12].

#### 1.2.3 Screening Technology Leading to Treatment

A multiplexed cell line screening platform combining a DNA‐barcoding technique and a Luminex microsphere detection system, PRISM, was established [13, 14].

The original PRISM platform struggled with high‐throughput genetic interrogation. To overcome this, researchers established the rapid validation of the therapeutic value of a given target of interest across a large panel of human cancer cell lines using both CRISPR and pharmacological perturbations, a new PRISM‐based system, a Bristol Myers Squibb (BMS)‐PRISM platform combining CRISPR/Cas9‐mediated gene editing capability, and a DNA‐barcoding multiplexing technique. With the BMS‐PRISM system, a focused run with epidermal growth factor receptor (EGFR) and a full library run with KRAS as proof‐of‐principle studies were reported. PRISM technology has advanced cancer research by enabling the profiling of drug activity on cell viability across numerous cancer cell lines, identifying genotype‐specific vulnerabilities. Utilizing PRISM, 4518 drugs on 578 cell lines were assessed and suggestions were made on which nononcology drugs could be repurposed for cancer treatment [15]. In addition, the current Depmap contains the data sets of PRISM.

Generally, a genome‐wide CRISPR screening is applicable in xenograft models. Ideally, tissue‐specific genomic perturbation In Vivo is expected. In this respect, the *Sleeping Beauty* transposon screening system still has an advantage In Vivo because tissue‐specific perturbation and tumorigenesis can be induced in mice with Cre‐loxP system and tissue‐specific Cre expression [3].

#### 1.2.4 Combining CRISPR Screening With Single‐Cell Analysis

Previous approaches involve the bulk transduction of cells with a gRNA library, with the subsequent analysis of gRNA distribution occurring before and after selective pressure. Commonly assessed phenotypes include variations in cell fitness under drug selection and changes with indicating specific markers. In recent years, single‐cell CRISPR screening techniques have been developed to address these challenges, allowing for a more detailed examination of the molecular consequences of genetic perturbations at the single‐cell level. These approaches combine CRISPR‐based perturbations with single‐cell analysis, facilitating the investigation of mutation–function relationships with greater resolution. Notably, methods such as single‐cell RNA sequencing (scRNA‐seq) integrated with CRISPR screening have been instrumental in elucidating these relationships. Additionally, advancements in the field have incorporated pooled perturbation techniques with high‐dimensional analyses, such as scRNA‐seq and mass cytometry for protein detection, enabling the capture of more intricate details about cell states and the effects of perturbations. Here, we introduce several modified CRISPR screenings combined with single‐cell analysis (see Table 2).

**Table 2:** Genetic screening methods that combine perturbation screening based on the CRISPR libraries with single‐cell analysis.

Sequence types	Basic principle	Advantage	Reference
Perturb‐seq	CRISPR/Cas9‐based gene perturbation + scRNA‐seq with transcriptional barcodes	High‐throughput, scalable analysis of genetic interactions and gene regulatory networks	Dixit et al. [16]
CROP‐seq	CRISPR/Cas9‐based gene perturbation + scRNA‐seq with a direct gRNA reading method		Datlinger et al. [17]
CITE‐seq	Detection of surface protein + scRNA‐seq	Profiling both gene and protein expression	Stoeckius et al. [18]
Perturb‐CITE‐seq	Perturb‐seq + CITE‐seq	Simultaneous analysis of transcriptomic and proteomic changes under specific genetic modifications	Frangieh et al. [19]
Scifi‐RNA‐seq	Preindexing cells + droplet‐specific barcodes	Individual transcriptome identification by removing cell doublets	Datlinger et al. [20]
SPEAC‐seq	Droplet‐based cell coculture + microfluidic sorting based on fluorescent reporter circuits	Analyze cell–cell interaction	Wheeler et al. [21]

Perturb‐seq is a high‐throughput method combining CRISPR/Cas9‐based genetic perturbation with scRNA‐seq to link genetic perturbations with their transcriptional impacts on an individual cell basis. In Perturb‐seq, lentiviral vectors deliver gRNAs that direct Cas9 to specific loci for gene knockouts, with each gRNA uniquely identifiable by an associated barcode. This pooled‐screening approach allows the simultaneous interrogation of multiple genetic loci, enabling both single‐gene knockouts and combinatorial perturbations. After perturbation, cells undergo scRNA‐seq, which captures messenger RNA (mRNA) and gRNA barcodes, allowing the identification of the transcriptional outcomes specific to each genetic modification. Perturb‐seq has been instrumental in studying complex gene regulatory networks, epistatic interactions, and transcriptional programs in diverse cell types. The technique's scalability is enhanced by adjusting the MOI, allowing systematic analyses across numerous genes. Computational tools such as MIMOSCA, multiple‐input multiple‐output single‐cell analysis, facilitate multidimensional analysis by linking transcriptomic changes to cellular states and functional pathways. Eventually, Perturb‐seq has been successfully applied to the study of immune activation in individual dendritic cells, identifying transcription factors regulating with lipopolysaccharide in each cell level, as well as novel regulators of differentiation, antiviral response, and mitochondrial function. Despite its scalability, systematic exploration remains challenging in Perturb‐seq [16].

At the same time, an alternative screening method, CRISPR droplet sequencing (CROP‐seq), has been reported. While Perturb‐seq uses transcriptional barcodes in single‐cell CRISPR screening, CROP‐seq uses a direct gRNA reading method. In CROP‐seq, leveraging the concept that gRNAs and their associated cellular responses are inherently compartmentalized within individual cells. By detecting each cell's gRNA alongside its single‐cell transcriptome, this approach enables the identification of gene expression signatures corresponding to specific gene knockouts within a heterogeneous population. The CROP‐seq method operationalizes this idea by integrating four critical components: a gRNA vector detectable in scRNA‐seq; a high‐throughput scRNA‐seq assay; a computational pipeline for linking transcriptomes to gRNAs; and bioinformatic tools for analyzing transcriptional changes. CROP‐seq has been applied to the study of individual T‐cell receptor (TCR) activation, identifying key regulators of TCR signaling, and uncovering the effects of negative regulators on TCR activation [17].

Cellular indexing of transcriptomes and epitopes by sequencing (CITE‐seq) is a technique that integrates scRNA‐seq with the simultaneous detection of surface protein levels using oligonucleotide‐labeled antibodies. This method overcomes the limitations of traditional scRNA‐seq, which lacks phenotypic information such as protein expression on the cell surface. CITE‐seq achieves this by conjugating antibodies to oligonucleotides, which are tagged with barcodes and can be detected through sequencing alongside transcriptomic data. This approach allows for the generation of multimodal data that include both transcriptomic and proteomic profiles of individual cells. It is particularly useful for immunophenotyping, where the expression of surface markers can distinguish different immune cell populations. This method has been shown to provide quantitative protein measurements comparable to flow cytometry: the gold standard for protein‐level analysis. Through integrated transcriptomic and protein marker analysis, CITE‐seq has identified subtle phenotypic distinctions such as CD56bright and CD56dim natural killer cell subsets and validated marker expression differences among T cells, B cells, monocytes, and dendritic cells. By integrating RNA and protein data, CITE‐seq enables a more detailed characterization of cell states and functions than either method alone, making it a valuable tool for studying complex cellular systems, such as the immune system, where both transcriptomic and proteomic information are critical for understanding cellular diversity and function [18].

Perturb‐CITE‐seq is an advanced extension of Perturb‐seq that combines scRNA‐seq with epitope sequencing (CITE‐seq) to analyze both transcriptomic and surface protein changes in cells following targeted genetic perturbations. Using single‐gRNAs linked to unique barcodes, this multimodal method captures gene perturbations at both the RNA and protein levels in individual cells, enabling a comprehensive view of cellular responses. Applied to melanoma cells cocultured with tumor‐infiltrating lymphocytes (TILs), Perturb‐CITE‐seq investigates genes associated with immune checkpoint inhibitor resistance across ~750 perturbations in 218 000 cells. Due to the technique's ability to simultaneously profile transcriptomes and surface proteins, in practice, Perturb‐CITE‐seq has been applied to the study of mechanisms of immune checkpoint inhibitor resistance in melanoma, identifying *CD58* loss as a novel factor that enables immune evasion independent of antigen presentation pathways. Perturb‐CITE‐seq offers a powerful framework for dissecting complex biological circuits, with significant implications for cancer immunotherapy and the study of multifactorial cellular processes [19].

Single‐cell combinatorial fluidic indexing RNA‐seq (scifi‐RNA‐seq) is a scalable, cost‐effective method for scRNA‐seq that significantly increases throughput by using combinatorial fluidic indexing. The core innovation of scifi‐RNA‐seq lies in its two‐step barcoding process, which allows multiple cells to be encapsulated in each droplet without losing the ability to distinguish their individual transcriptomes. First, cells are permeabilized, and their transcriptomes are preindexed with unique barcodes in a “split‐pool” setup. This initial indexing enables the labeling of RNA from each cell before they are pooled together. Subsequently, the preindexed cells are loaded into a microfluidic droplet generator, where they are encapsulated in droplets with additional barcodes specific to each droplet. The combination of preindexing and droplet‐specific barcoding ensures that the transcriptomes from individual cells can be computationally demultiplexed, even when multiple cells share the same droplet.　Scifi‐RNA‐seq offers several advantages, including improved efficiency and cost‐effectiveness by allowing higher cell concentrations in each droplet. This method is particularly useful for large‐scale applications, such as cell atlas projects or high‐throughput perturbation screens. Finally, scifi‐RNA‐seq has been applied in a highly multiplexed CRISPR screen for TCR activation, uncovering key regulators of T cell activation and providing insights into the underlying transcriptomic changes associated with immune response. By overcoming the limitations of conventional droplet‐based scRNA‐seq, scifi‐RNA‐seq facilitates the analysis of millions of single cells in a single experiment, making it an ideal tool for large‐scale single‐cell studies [20].

Recent advancements in high‐throughput microfluidics‐supported genetic screening have facilitated the discovery of functional regulators of cell–cell interactions through a technique known as systematic perturbation of encapsulated associated cells followed by sequencing, SPEAC‐seq. The technique integrates CRISPR‐based genetic perturbations with microfluidic coculture of cells in picoliter droplets, followed by fluorescence‐activated droplet sorting. In SPEAC‐seq, two cell types are coencapsulated in droplets, with one cell harboring a CRISPR‐induced genetic perturbation and the other expressing a fluorescent reporter. The reporter cell signals functional outcomes of the interaction, such as NF‐κB activation in response to inflammation. After coculture, droplets showing reporter activation are enriched and sequenced, allowing identification of the genetic regulators of cell–cell interactions. SPEAC‐seq has been applied to the study of neuroinflammatory communication between microglia and astrocytes, resulting in the discovery of regulators of astrocyte NF‐κB activation [21]. It can also be adapted for other cell types and biological processes, such as T cell activation or antigen presentation. The platform's scalability allows for genome‐wide screens, enabling the discovery of novel regulators of intercellular communication at a large scale. However, SPEAC‐seq is constrained by the availability of fluorescent reporters to signal functional outcomes of cell–cell interactions. Moreover, the in vitro setting of microfluidic droplets may not fully replicate physiological cell environments, leading to nutrient limitations and suboptimal culture conditions. The technical complexity and cost of the required microfluidic and optical systems may also limit its accessibility for widespread use [21, 22].

#### 1.2.5 Revealing the Spatial Interaction of Cells in Microenvironments

**Figure 3:**
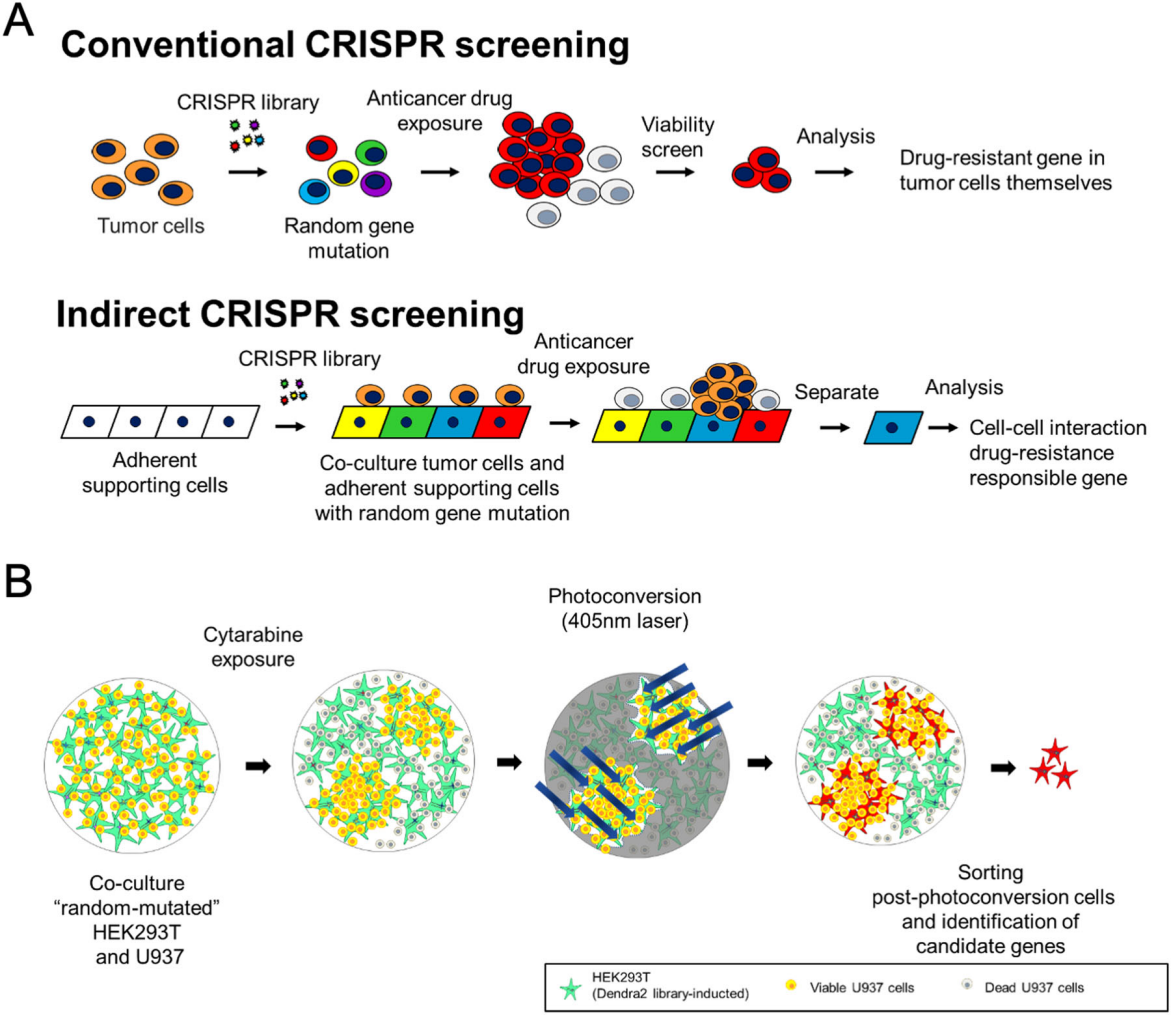
Schemes of conventional and indirect CRISPR screening. (A) The experimental model. In conventional CRISPR screening, the candidate genes for drug resistance are identified by detecting the increasing/decreasing number of tumor cells with guide RNAs (gRNAs) inducing random mutations under screening conditions (upper tier). Meanwhile, in indirect CRISPR screening, random mutations are inducted into the adherent supporting cells and cocultured with the tumor cells to create so‐called “microenvironments.” The responsible supporting cells are then separated and analyzed for drug resistance with cell–cell interactions (lower tier). (B) Indirect CRISPR screening system with Dendra2. HEK293T and U937 cells were inducted as cell–cell interaction systems to mimic the microenvironment. U937 was cocultured with HEK293T inducted with Dendra2 and the CRISPR library. Under cytarabine exposure, most U937 cells were killed, and only some U937 cells close to HEK293T survived and proliferated. The supporting cells that induced drug resistance in tumor cells were expanded and then identified by laser scanning microscopy. They then underwent photoconversion with the 405 nm laser. After photoconversion, the red‐fluorescing HEK293T cells were sorted on FACS. Sorted cells were analyzed to identify the genes responsible for drug resistance induced by cell–cell interactions. *Source:* Adapted from Sugita et al. [23], with minor modifications.

In cases of drug resistance induced by cell–cell interactions from the surrounding environment, such as tumor microenvironments (TME), identifying the candidate genes responsible for drug resistance associated with these interactions is challenging. This difficulty arises because there is no selective increase or decrease in the number of surrounding cells, which limits the utility of conventional CRISPR screening that identifies candidate genes based on the selective proliferation or death of cells (Figure 3A).

To explore the molecules responsible for drug resistance induced by peritumoral cell–cell interactions, a new CRISPR screening system, indirect CRISPR screening, was established in our previous study. In indirect CRISPR screening, random mutations are inducted into the adherent supporting cells and cocultured with the tumor cells without mutation to mimic TMEs. The responsible supporting cells are then separated and analyzed for drug resistance with cell–cell interactions. To isolate them, using responsible supporting cells, we induct Dendra2 as the labelable fluorescent marker to supporting cells. In the screening experiments, Dendra2 was inducted to supporting cells and the drug resistance responsible factors of supporting cells were explored with CRISPR screenings (Figure 3B).

To mimic the TME, HEK293T cells were inducted as supporting cells and U937 leukemia cells were inducted as tumor cells, which were easy to handle technically, into cell–cell interaction systems. Dendra2‐inducted HEK293T cells, with the CRISPR knockout library also inducted, were cocultured with U937 cells. U937 is relatively more sensitive to cytarabine than HEK293T, and coculture screening was performed with cytarabine which can kill most U937 cells to observe outstanding‐living U937 cells. Most U937 cells were killed by cytarabine. As we expected, a small number of U937 in close proximity to HEK293T survived and proliferated. All supporting cells close to the viable U937 colonies under cytarabine exposure were applied to photoconversion. The supporting cells that induced drug resistance near viable U937 colonies were identified by differential interference images from laser scanning microscopy, and photoconversion was performed with a 405 nm laser. After photoconversion, the red form of Dendra2 was confirmed with laser scanning microscopy (559 nm laser) and the PI filter of FACS. Then HEK293T cells with the red form of Dendra2 were sorted from each well by FACS. Collected mutated HEK293T were analyzed using TA cloning, and 39 candidate genes were obtained. These 39 candidate genes were validated to determine whether they could reproducibly induce drug resistance and associated mechanisms. Eventually, *Rhbdd2* was identified as a gene responsible for drug resistance with cell–cell interactions. This gene screening system, which focuses on labeling and isolating living phenotype‐inducible mutant cells, not selectively grown tumor cells, under microscopic observation, has not been reported previously. Therefore, this method could be useful to reveal unknown mechanisms behind a variety of cell–cell interactions and collecting living cells is also a significant advantage of biological analysis. It is also a potential platform for discovering the new targets of drugs in combination with conventional chemotherapy [23].

Perturb‐map is a spatial genomics platform that integrates CRISPR screening with Visium spatial transcriptomics and multiplex imaging, providing powerful tools for gene function analysis in intact tissue. By using protein barcoding (Pro‐Code), Perturb‐map uniquely labels CRISPR‐edited cell populations, enabling In Vivo assessment of gene impacts on the TME while maintaining spatial architecture. This approach supports the simultaneous knockout of multiple genes, elucidating gene‐specific influences on tumor growth, immune composition, and histopathology, particularly by illustrating how certain genes regulate immune cell localization and activity within the TME. In a practical application, Perturb‐map enabled the simultaneous knockout of up to 35 genes in a mouse lung cancer model, revealing specific influences on tumor growth and immune response. For instance, *Tgfbr2* knockout led to a fibro‐mucinous TME with T cell exclusion, underscoring the role of TGFβ signaling in immune landscape modulation. Conversely, *Socs1* knockout promoted T cell infiltration, highlighting its regulatory role in immune response. This platform represents a significant advancement in spatial CRISPR applications, offering new pathways for therapeutic strategies in cancer research. However, Perturb‐map's CRISPR library size remains smaller than traditional in vitro screens, constrained by model limitations, as the number of feasible tumor lesions restricts library capacity. Additionally, highly heterogeneous tumors may support larger libraries but are less suitable for studying extracellular gene functions, emphasizing the importance of careful model selection. Moreover, it is noteworthy that this system primarily screened for random mutations in tumor cells and subsequently analyzed the expression profiles of stromal cells, without initially screening for random mutations in the stromal cells themselves [24].

Visium, an established spatial transcriptomics tool, offers whole‐transcriptome coverage, allowing for broad spatial characterization and de novo identification of regions or cell types within tissues. This unbiased approach makes Visium particularly useful for exploratory studies where cellular diversity across large tissue regions is of interest. In contrast, the recently developed Xenium by 10x Genomics provides a highly targeted approach that focuses on a set of 5000 genes, with options to add up to 100 additional targets [25, 26, 27]. This customizable panel is especially advantageous for studies focused on specific genes within complex tissue with cellular diversity in restricted regions, such as immune and myoepithelial cells. However, unlike Visium, Xenium lacks the broad discovery capability necessary for unbiased spatial characterization, limiting its applicability in exploratory research.

#### 1.2.6 CRISPR Screening In Vivo

Recent studies have reported the use of In Vivo genome‐wide CRISPR screening with preclinical models that more accurately mimic the 3D TME of patients [28, 29, 30]. Although such screens have been conducted in lung cancer, leukemia, and breast cancer, their effectiveness in addressing unmet medical needs remains unestablished, highlighting the need for further investigation into their practical clinical applications. Additionally, recent research has examined immune evasion mechanisms across multiple cancer types, including melanoma, pancreatic, lung, renal, colon, and breast cancers, using genome‐scale In Vivo screens [31, 32]. The use of advanced models, including organoids and humanized mice, promises to yield more physiologically relevant insights, driving forward our understanding of complex biological systems. Generally, a genome‐wide CRISPR screening is applicable in xenograft models. Ideally, tissue‐specific genomic perturbation In Vivo is expected. In this respect, the Sleeping Beauty transposon system still has an advantage.

#### 1.2.7 Development Screening With Alternative Cas Proteins

Successful CRISPR screening relies on efficient Cas9 and gRNA delivery, particularly in primary cells and In Vivo. Optimizing gene perturbation, model selection, and stimuli calibration is essential for capturing relevant biological processes accurately [33]. Recent screening advancements have moved beyond Cas9, with alternative Cas proteins improving precision and versatility. While using Cas9 can lead to multiple double‐strand breaks, recent methods leverage transcriptional (CRISPRi) or RNA‐level targeting to reduce these events [34]. For instance, Cas13 RNA Perturb‐seq (CaRPool‐seq) employs Cas13 for targeted knockdown at the transcript level [34, 35]. Additionally, Cas12 offers the advantage of processing multiple guides from a single transcript, allowing significant reductions in vector and library size and supporting CRISPR knockout, CRISPRa, and CRISPRi applications [34, 35, 36, 37, 38, 39]. These refined methods enable the precise assessment of gene expression effects by targeting coding and noncoding (regulatory) regions, facilitating more comprehensive genetic perturbation studies [34].

## 2 Future Perspective

Conventional use of CRISPR screening is still a powerful tool for clarifying the mechanism of interest and identifying its key molecules. Its operation has become significantly easier. There is no need for anyone to hesitate to use it.

To achieve more advanced and effective screenings, it is necessary to clarify the significant gaps that often remain between identifying causal effects and elucidating the underlying biological events [34]. Extracting meaningful biological insights from Perturb‐seq screens requires advanced computational approaches, as they capture global, cell‐level impacts of genetic perturbations across a high‐dimensional phenotypic space. Unlike traditional pooled screens, which rank single‐feature hits, Perturb‐seq screens reveal complex interactions between perturbations and diverse cellular features, allowing researchers to map regulatory mechanisms at multiple levels. This broader view enables the identification of gene programs (groups of coregulated genes), regulatory modules, and cofunctional perturbation modules, creating a comprehensive representation of cellular responses. Mechanistic models aim to bridge these gaps by integrating perturbation screens with mechanistic data derived either from the screen's own outputs or from auxiliary sources such as binding, proteomics, phosphorylation, or other complementary data sets. Further integration of engineering and computational methods is needed to improve precision and mechanistic depth in CRISPR‐based studies [34].

Recent innovations in CRISPR technology have also introduced regulatory and epigenetic manipulation techniques that further enhance the exploration of complex cell states and pathways [33]. The development of spatial transcriptomics technologies, represented by Visium, has contributed significantly to comprehensive and high‐content screening In Vivo by enabling high‐resolution transcriptomic profiling across various tissues. However, such high‐content screening microscopes allow detailed mechanistic studies, though they come at a high cost, which may limit their compatibility with diverse sample types and their integration with other automation systems [40]. These limitations underscore the need for more accessible and cost‐effective options to perform comprehensive cellular and genetic explorations. Similarly, our indirect CRISPR screening approach targets In Vivo applications and utilizes photoconversion to identify target cells within living organisms in the future [23].

Our literature review suggests that none of the drug targets identified in the presented study have yet been implemented in clinical oncology practice. This is because the development of new compounds takes time. Nevertheless, we anticipate that future studies will be able to connect the biological mechanisms, revealed by the screenings, presented in this manuscript to drug discovery efforts and validate the efficacy of each candidate drug. We remain hopeful that these agents will eventually be incorporated into clinical oncology practice.

## Author Contributions

**Keisuke Sugita:** drafting of the manuscript and figures. **Morito Kurata:** drafting of the manuscript and figures. Both authors approved the final manuscript.

## Conflicts of Interest

The authors declare no conflicts of interest.
